# Correction: Tamoxifen Response at Single Cell Resolution in Estrogen Receptor-Positive Primary Human Breast Tumors

**DOI:** 10.1158/1078-0432.CCR-25-1944

**Published:** 2025-07-01

**Authors:** Hyunsoo Kim, Austin A. Whitman, Kamila Wisniewska, Rasha T. Kakati, Susana Garcia-Recio, Benjamin C. Calhoun, Hector L. Franco, Lisa A. Carey, Charles M. Perou, Philip M. Spanheimer

In the original version of this article (1), author Lisa A. Carey was mistakenly omitted from the author list. The error has been corrected in the latest online HTML and PDF versions of the article and the author contributions and disclosures have been updated as necessary. The authors regret the error.
